# Perceptual differences in nursing implementation leadership and climate: a cross-sectional study

**DOI:** 10.1186/s43058-023-00392-9

**Published:** 2023-01-20

**Authors:** Clayton J. Shuman, Mark G. Ehrhart, Philip T. Veliz, Marita G. Titler

**Affiliations:** 1 School of Nursing, University of Michigan, 400 N. Ingalls, Room 4162, Ann Arbor, MI 48109 USA; 2 Institute for Healthcare Policy and Innovation, University of Michigan, Ann Arbor, MI USA; 3 Center for the Study of Drugs, Alcohol, Smoking, and Health, University of Michigan School of Nursing, Ann Arbor, MI USA; 4 Department of Psychology, University of Central Florida, Orlando, FL USA

**Keywords:** Nursing administration research, Nursing staff, Hospital, Leadership, Climate, Implementation, Evidence-based practice, Practice context

## Abstract

**Background:**

The literature on perceptual differences between managers and staff regarding social dynamic factors (e.g., leadership, climate) in nursing settings is sparse. Addressing this gap in knowledge is critical for informing implementation efforts and improving patient and organizational outcomes. The purpose of this study was to test the perceptual differences regarding implementation leadership and implementation climate between nursing staff and their managers.

**Methods:**

This study was a secondary analysis of cross-sectional survey data collected in 2016–2017. The setting included 22 adult medical-surgical units nested in 7 acute care hospitals in the Eastern and Midwestern United States. Participants were registered nurses (*N* = 261) and nurse managers (*N* = 22) who completed an electronic survey consisting of the Implementation Leadership Scale (ILS), the Implementation Climate Scale (ICS), and demographic items. Differences in perception were analyzed at the unit level using structural equation modeling to develop latent difference score models (LDS). We assessed associations of the LDSs with manager ILS and ICS scores, years of nursing experience, and years of experience working on the current unit. The association of ILS LDS with the observed nursing staff ICS scores was also analyzed.

**Results:**

Higher manager scores on the ILS and ICS were associated with greater perceptual differences in implementation leadership and implementation climate. Greater years of experience as a nurse were associated with greater perceptual differences in ILS and ICS scores. Greater tenure on the unit was associated with smaller differences on the ILS knowledge domain. Greater perceptual differences regarding implementation leadership were associated with worse staff ratings of implementation climate.

**Conclusions:**

Although this study observed significant relationships among manager ILS and ICS scores, staff-manager perceptual differences, and staff ratings of implementation climate in nursing settings, it is still unclear why perceptual differences in implementation leadership and climate exist and how to address them. Future studies are warranted to test the effect of perceptual differences on implementation and patient outcomes.

**Supplementary Information:**

The online version contains supplementary material available at 10.1186/s43058-023-00392-9.

Contributions to the literature

- This study addresses gaps in knowledge about the implementation context in nursing settings and identified misalignment in nursing staff and management perceptions of unit-level implementation leadership and climate.
- Implementation climate is an important contributor to implementation outcomes and is influenced by a unit leader’s behaviors. Results from this study suggest alignment in perceptions of implementation leadership is associated with more conducive implementation climates.
- Nurse managers maintain responsibility for supporting implementation efforts; however, staff nurses may not be receiving the support necessary for successful implementation.

## Background

A critical issue in healthcare is how to most effectively and efficiently translate the evidence on effective and high-quality patient care into practice. Evidence-based practices (EBPs) are those that have demonstrated efficacy and effectiveness and may include programs, treatments, interventions, procedures, processes, policies, or guidelines [1]. To achieve the gains EBPs can provide, they must be implemented effectively. In line with the literature on the implementation of innovations in the management and industrial/organizational psychology literatures [2, 3], the implementation of EBPs in healthcare settings is critical to providing high-quality patient care and achieving optimal patient and organizational outcomes [4]. However, translating EBPs into routine practice continues to be a significant challenge for implementation scientists, quality improvement professionals, and, more broadly, hospitals and healthcare systems [5, 6]. Numerous studies have demonstrated that implementation is challenged by a multitude of factors, many of which are related to the context or setting where implementation occurs [4, 7–9].

In the nursing literature, the context where implementation takes place emphasizes two primary features: structural factors and social dynamic factors [10]. Structural factors refer to key characteristics of the setting such as staffing models, type of units or patients, and unit size. Social dynamic factors refer to the roles and relationships among individuals and groups within the context. Many studies support assessing the context prior to implementation and targeting implementation strategies to reinforce positive and mitigate negative aspects of the climate that affect implementation [11–13]. Although studies have examined the structural factors affecting implementation and quality improvement, less attention has been given to the social dynamic factors, specifically the leadership and unit climate for implementation [14, 15].

Leadership is considered an important social dynamic factor of context that facilitates and/or impedes implementation efforts [10, 15–18]. As frontline leaders of nursing units, nurse managers maintain numerous managerial responsibilities, including but not limited to, budgeting, hiring staff, overseeing patient care, and providing EBP resources for staff [19]. Thus, they are ideally positioned to lead implementation efforts on their units to improve care delivery and health outcomes [20]. Castiglione [21] defined implementation leadership as “a specific and strategic approach to leadership characterized by a set of influencing behaviors leading to positive outcomes for the implementation of EBPs” (p. 94). The set of influencing behaviors and characteristics managers enact to lead EBP implementation include (1) achieving EBP competency (e.g., able to answer staff’s questions about EBP); (2) being proactive about implementing EBPs (e.g., developing a plan for implementation); (3) providing support and resources to help staff implement EBPs (e.g., training); and (4) persevering through implementation challenges (e.g., identifying solutions to implementation barriers) [15, 16].

One of the primary mechanisms through which leaders have an impact on unit-level outcomes is through the climate they develop in their units [22]. Implementation climate is described as a strategic climate supportive of implementation that is facilitated by the practices, policies, and procedures that are expected, supported, and rewarded [2, 14, 23, 24]. Positive climates for EBP implementation are associated with improved implementation outcomes (e.g., adoption; sustainability) as well as patient and organizational outcomes [25–27]. Nurse managers play a critical role in facilitating unit climates that are more conducive to EBP implementation [10, 15, 28].

The relationship between implementation leadership of managers and the unit climate for implementation has been described in both nursing [10] and non-nursing settings [27, 29]. Yet, little is known about the differences between managers and their staff in their perceptions of these context factors. Although Aarons et al. [30] found that perceptual differences between managers and staff were related to the climate of their unit, more research is needed to understand the implications of such perceptual differences for the social dynamic relationships among nurse managers and staff nurses that are important to implementation. In addition, understanding how perceptual differences in leadership are related to unit climate for implementation can inform intervention work targeting these social dynamic factors. Therefore, the purposes of this study were to (1) evaluate the extent to which nurse managers and nursing staff differed in their perceptions of implementation leadership and implementation climate; (2) examine the association of manager perceptions with the difference between manager and staff perceptions of implementation leadership and implementation climate; (3) explore the associations among staff characteristics and the perceptual differences; and (4) evaluate the association between perceptual differences in implementation leadership and the unit climate.

### Theory and hypotheses

When considering the relative level of implementation leadership and climate scores for nurse managers comparative to their nursing staff, we expected that scores on both constructs would be generally higher for managers than staff. There are two primary theoretical explanations from research to support this hypothesis. The first is from the literature on self-serving biases—specifically, the Dunning-Kruger effect [31]. In general, the Dunning-Kruger effect is based on the tendency for individuals to rate themselves “above average” despite their actual level of ability or performance. One explanation for this effect is that individuals performing low in a certain domain carry a double burden of not only performing poorly but also not knowing what good performance is and thus being unable to recognize it [32].

A second theoretical explanation for expecting managers to rate implementation leadership and climate higher than staff is based on the accessibility of information. Specifically, leaders are more familiar with the totality of their overall behavior than their subordinates and, thus, staff may not be aware of the extent to which the leader actually emphasizes implementation in the totality of their interactions. This explanation has been used to explain why correlations among sources of performance ratings are often low (in addition to the explanation of rater bias); because of this greater access to relevant information, leaders will be more likely to know about their own implementation efforts than will staff [33].

Although the above explanations focus on ratings of the leader’s own behavior (e.g., implementation leadership), we assert that the same logic would apply when considering implementation climate. Implementation climate is based on perceptions of the policies, practices, and procedures that support implementation [14]. Managers are typically responsible for developing and implementing the policies, practices, and procedures that are the targets of climate ratings [22, 34, 35]. Therefore, nurse managers may be affected by self-serving biases when rating the implementation climate of their unit because the unit will be viewed as an extension of themselves, and thus they will be motivated to view the climate of the unit in a positive light. Furthermore, they are likely to be more aware than staff regarding the policies, practices, and procedures put into place, and/or the extent to which these policies, practices, and procedures support implementation in the larger organization. Empirical research from both the early organizational climate literature [36] and the nursing literature [37] supports the assertation that leaders will be more likely to rate climate higher. Therefore, we made the following hypothesis:

*Hypothesis 1:* Nurse managers will have higher scores on implementation leadership (H1a) and implementation climate (H1b) relative to nursing staff across all units.

The second hypothesis focuses on the relationship between the manager scores on implementation leadership and implementation climate and perceptual differences with staff. Aarons and colleagues [30] found that perceptual differences among leaders and staff regarding implementation leadership were associated with strategic organizational climates for involvement and performance feedback. In their study, organizational climate was highest (best) when leaders scored themselves low on implementation leadership and their staff scored them high. In this study, it is anticipated that managers’ scores will be generally higher than staff scores on implementation leadership and climate and that the higher manager ratings are, the more likely the managers are not in touch with the reality of their units (i.e., the larger the perceptual differences). This hypothesis is in line with research on the Dunning-Kruger effect across a variety of domains, which has found that even though high-performing individuals can underestimate their actual performance levels, the largest gaps are found between low-performing individuals’ self-ratings and their actual performance levels [32]. Thus, we hypothesize the following:

*Hypothesis 2:* Higher nurse manager ratings of implementation leadership (H2a) and implementation climate (H2b) will be associated with greater perceptual differences relative to staff.

Next, we hypothesized that staff experience is associated with smaller gaps in perceptions of implementation leadership and climate relative to managers. This hypothesis is directly tied to access to information to explain differences between ratings of managers and staff. Two types of experience are of interest: overall experiences as a registered nurse (RN) and tenure on the current unit. As staff gather more overall nursing experience, it is anticipated that they will increase their understanding of leadership and climate, in addition to being more involved, potentially, in unit initiatives and implementation efforts. Essentially, they are more likely to act as informal leaders [38, 39], and as such, would be privy to similar types of information as that of the managers. As a result, higher-tenured staff’s perspectives on implementation leadership and implementation climate should be more aligned with their manager’s perspective. The reasoning for tenure on the unit is similar, although this variable captures experience specifically relevant to the manager’s and the policies, practices, and procedures in that unit, all of which should further contribute to more aligned perceptions with the manager. Thus, we hypothesize the following:

*Hypothesis 3:* More years of staff experience as a RN will be associated with smaller perceptual differences between managers and staff on implementation leadership (H3a) and implementation climate (H3b).

*Hypothesis 4:* More years of staff experience on the current unit for RNs will be associated with smaller perceptual differences between managers and staff on implementation leadership (H4a) and implementation climate (H4b).

Finally, we examined the effects of alignment of perceptions of implementation leadership on implementation climate. Because it is anticipated that managers will generally rate themselves higher on implementation leadership than staff, we assumed that when there is alignment, it will generally occur around higher levels of implementation leadership. When managers report that they are performing many implementation-related activities, but staff do not, then that indicates that the manager either is highly biased in their self-ratings in line with the Dunning-Kruger effect, or that they are actually performing implementation behaviors and instituting policies and procedures related to implementation, but their implementation efforts are having little impact on the staff. For either of these reasons, implementation climate perceptions should be relatively lower, in line with past research showing the negative influence of over-estimators [40, 41]. However, when managers and staff view implementation leadership similarly, that indicates a better-functioning unit and more awareness of the manager’s implementation efforts by staff, which should result in higher implementation climate.

*Hypothesis 5:* Greater perceptual difference between staff and managers in implementation leadership will be associated with lower ratings of implementation climate by staff.

## Methods

### Design

Data for this secondary analysis were collected as part of a larger study interested in describing the practice context for EBP implementation in nursing settings. The study was originally conducted in 2016–2017 and used a cross-sectional descriptive design [10, 42].

### Participants

The participants included (1) nurse managers and (2) staff nurses caring for patients from seven hospitals in the Midwest and Northeast United States. Three hospitals were small (<100 beds), two were medium (100–300 beds), and two were large (>300 beds). Six hospitals were private and not-for-profit, four were church-affiliated, and two had current Magnet® designation. Study units (*N* = 22) from each hospital met the following criteria: (1) cared for adult patients; (2) designated as a medical, surgical, or medical-surgical unit; and (3) had a nurse manager who met inclusion criteria. If one manager oversaw multiple eligible units, one of their units was randomly selected for inclusion. Units varied in size (9–45 patient beds) and staffing skill mix (proportion of RNs to assistive personnel; mean= 60%, SD= 10%).

Nurse managers met the following inclusion criteria: (1) licensed as a registered nurse; (2) had responsibility and accountability for unit-level operations; (3) not in an interim role; and (4) was direct supervisor of nursing staff on the study unit. Staff nurses (1) were licensed as registered nurses, (2) worked a minimum of .40 full-time equivalents, (3) provided direct patient care, and (4) were designated as staff on the study unit. Thirty eligible staff nurses per unit were randomly selected to receive invitations to participate. For units with less than thirty eligible staff nurses, all eligible were invited to participate.

The final sample included 261 staff and 22 managers. This secondary analysis used 98.9% of staff and 100% of manager responses collected during the original study. Records with missing data were listwise deleted. The original study had a 91.7% nurse manager response rate and a 51.9% staff nurse response rate [10]. Overall, staff had a mean age of 35.52 years (SD=11.98), mean number of years of RN experience of 8.16 (SD=10.02), and mean number of years of RN experience on their current unit of 5.13 (SD=7.46). When aggregated to the unit level for analysis, the mean number of years of RN experience was 8 (SD=3.56) and mean number of years on their current unit was 4.66 (SD=2.22). Fifty-six percent of staff had a bachelor’s degree in nursing and 31% had an associate’s degree. Nurse managers had a mean age of 41.76 (SD=6.67). Fifty-five percent of nurse managers had a bachelor’s degree in nursing and 32% had an associate’s degree. Both samples were predominantly white (82–86%, respectively).

### Study variables and measures

#### Implementation leadership

Implementation leadership is defined as the specific leadership behaviors enacted by nurse managers to facilitate EBP implementation and was measured using the 12-item *Implementation Leadership Scale* (ILS) [16]. The ILS includes four domains (1) proactive leadership (e.g., “My manager has developed a plan to facilitate implementation of EBPs”); (2) knowledgeable leadership (e.g., “My manager is able to answer my questions about EBP”); (3) supportive leadership (e.g., “My manager supports nurses’ efforts to learn more about EBP”); and (4) perseverant leadership (e.g., “My manager carries on through the challenges of implementing EBPs”). Respondents indicate their level of agreement with each item using a 0 (not at all) to 4 (very great extent) scale. The ILS has demonstrated reliability and validity in nursing settings [43]. Total and subscale scores were calculated by averaging the scores on the appropriate items. Staff completed the ILS, rating their respective managers. For this study, sufficient agreement was observed to aggregate scores to the unit level (r_WG(j)_ range= .88–.98). Managers completed the scale as a self-assessment.

#### Implementation climate

Implementation climate is defined as the shared perception of what is expected, rewarded, supported, and recognized regarding implementation of EBPs in the unit and was measured using the 18-item *Implementation Climate Scale* (ICS) [14]. The ICS consists of six domains: (1) unit focus on EBPs (e.g., “Using EBP is a top priority in my unit”); (2) educational support available for EBPs (e.g., “My unit provides EBP trainings or in-services”); (3) recognition for using EBPs (e.g., “Nurses who use EBPs are held in high esteem in this unit”); (4) rewards for using EBPs (“The better you are at using EBPs, the more likely you are to get a bonus or raise”); (5) hiring staff who value EBP (e.g., “This unit hires staff who have previously used EBPs”); and (6) hiring staff open to implementing new EBPs (e.g., “This unit hires staff who are open to new types of innovations”). Respondents indicate their level of agreement with each item on a 0 (not at all) to 4 (very great extent) scale. Reliability and validity for the ICS have been demonstrated in nursing settings [23]. Total and subscale scores were calculated for each manager and staff by averaging the scores on the appropriate items. In this study, sufficient agreement was observed to aggregate staff nurse scores to the unit level (r_WG(j)_ range= .83–.98).

### Study procedures

Site coordinators at each study site identified eligible participants and sent the organization affiliated email addresses to the study team. The study team randomly selected 30 eligible staff participants from each unit to receive an email invitation to participate. A similar email was sent to managers. A link in the email directed the participants to a manager or staff web-based survey, respectively. The survey included study information, selected demographic items, and the ILS and ICS. Participants were asked to complete the survey at their own convenience within 1 month. Weekly reminders to complete the survey were sent by the study team to participants’ work email addresses. After completing the survey, participants were invited to enter a raffle drawing for a $100 gift card.

### Data analysis

To test the distinction between the constructs of implementation leadership and implementation climate, we conducted a confirmatory factor analysis of staff-reported ILS and ICS measures. Results showed that the two-factor solution had acceptable fit (CFI= .932; TLI= .910; RMSEA= .211, *p*<.001) and fit significantly better than a one-factor solution (*χ*^2^= 72.3 (1), *p*<.001), supporting the distinction between these constructs in the implementation research literature. Descriptive statistics for the ILS and ICS scores among managers and staff across units were examined first. Descriptive statistics were also calculated for the crude difference scores (e.g., unit level staff score minus manager score) between managers and staff for ILS and ICS total and subscale scores. Additional analyses assessed correlations among ILS, ICS, and demographic characteristics across units (Supplemental file 1). Moreover, with respect to the correlations between manager and staff ILS and ICS scores, significant moderate positive correlations were found between managers’ ILS and ICS scores and between staff ILS and ICS scores. In general, no statistically significant association was found between managers’ ILS and ICS scores and staff ILS and ICS scores; however, these scores were negatively associated between managers and staff.

Next, structural equation modeling was used to develop latent difference score models (LDS) [44, 45] to optimize the reliability of the differences in ILS and ICS scores between managers and staff. LDS partitions the observed scores and measurement error to observe the difference between the two reliable parts. All latent difference analyses were conducted at the unit level. For the analysis, staff ILS and ICS were treated as *y*_1_ and manager ILS and ICS scores as *y*_0_ to estimate a reliable latent difference score (i.e., LDS_y1_ = *y*_1_ − *y*_0_). Accordingly, each LDS model assessed the association of observed unit-aggregated measures of manager ILS and ICS scores, age of staff, number of years of experience as an RN for staff, number of years working as a staff RN on their unit, and RN level of education with the predicted LDS for the ILS and ICS. Moreover, additional analyses assessed the association of ILS LDS with the staff and manager ICS scores. All models used full information maximum likelihood estimation to handle item missingness and adjust standard errors for clustering at the hospital level. All the estimated LDS models were just-identified models and yielded perfect fit as the focus was on the associations of the covariates on the LDS score, not model fit. Moreover, we also examined the results assessing the raw difference score (nursing staff − nurse manager) using Ordinary Least Squares regression (multiple linear regression) as a sensitivity check with respect to the robustness of our results from the LDS models; however, the results section below only focuses on the LDS models.

### Ethical approval

This study received ethical approval from the University of Michigan Institutional Review Board and the institutional review boards at each participating hospital. All participants were provided with a study information document which provided details about the study and their participation. Participants signified consent by completing and submitting their responses. Participants were assured their responses were confidential, their participation in the study would not be shared with their employer, and only anonymous and aggregated results would be disseminated.

## Results

Table 1 shows the descriptive statistics aggregated by role across units for managers and staff for the ILS, ICS, and difference scores. Hypothesis 1 stated that managers will have higher scores on implementation leadership (H1a) and implementation climate (H1b) relative to staff. For the ILS, H1a was supported for the subscale of supportive leadership (*p*<.05). In contrast, significantly higher staff scores were observed for the ILS subscale for knowledgeable leadership (*p*<.05). Total ILS score was not significant. For the ICS, H1b was not supported. ICS total score and subscales were not significantly different. The bivariate association between managers and staff scores (see Table 1) was inversely associated for the ICS subscale of educational support for EBP (*r*= −.493, *p*<.05) (e.g., as educational support for EBP scores increased for managers, scores for their staff decreased).

**Table 1:** Summary of aggregated perceptual differences on the ILS and ICS by unit (*N* = 22)

	Crude difference (staff minus manager) M (SD)	Crude difference spread Max, Min	Manager score M (SD)	Staff score M (SD)	*r*
**Implementation Leadership Scale (ILS)**					
Total scale	0.22 (0.73)	.970, −1.95	2.77 (0.40)	2.79 (0.52)	−.251
Proactive leadership	0.27 (.890)	1.55, −1.61	2.30 (0.65)	2.57 (0.50)	−.162
Knowledgeable leadership	0.33 (0.77)*	1.59, −1.87	2.57 (0.50)	2.90 (0.54)	−.099
Supportive leadership	−0.29 (0.78)*	0.89, −2.20	3.27 (0.51)	2.97 (0.49)	−.210
Perseverant leadership	−0.19 (0.99)	1.00, −2.13	2.93 (0.43)	2.74 (0.56)	−.268
**Implementation Climate Scale (ICS)**					
Total scale	−0.06 (0.85)	2.10, −0.98	2.22 (0.65)	2.16 (0.43)	−.208
Focus on EBP	−0.07 (1.02)	1.27, −2.60	2.66 (0.81)	2.58 (0.43)	−.308
Educational support for EBP	−0.08 (1.17)	1.92, −2.57	2.28 (0.86)	2.20 (0.46)	−.493*
Recognition for EBP	0.07 (1.10)	1.61, −2.06	2.24 (0.84)	2.31 (0.42)	−.215
Rewards for EBP	0.23 (1.01)	1.67, −2.57	1.09 (0.98)	1.32 (0.54)	.213
Selection for EBP	0.15 (1.09)	2.25, −2.26	2.01 (0.86)	2.16 (0.49)	−.218
Selection for openness	−0.29 (0.84)	1.06, −2.27	2.71 (0.69)	2.41 (0.46)	−.030

Cronbach’s alpha total scale: staff ILS = .998, manager ILS = .746, staff ICS = .963, manager ICS = .887

*EBP* evidence-based practice, *r* bivariate zero order correlation

*=*p*<.05 on one-tailed significance test

**Fig. 1:**
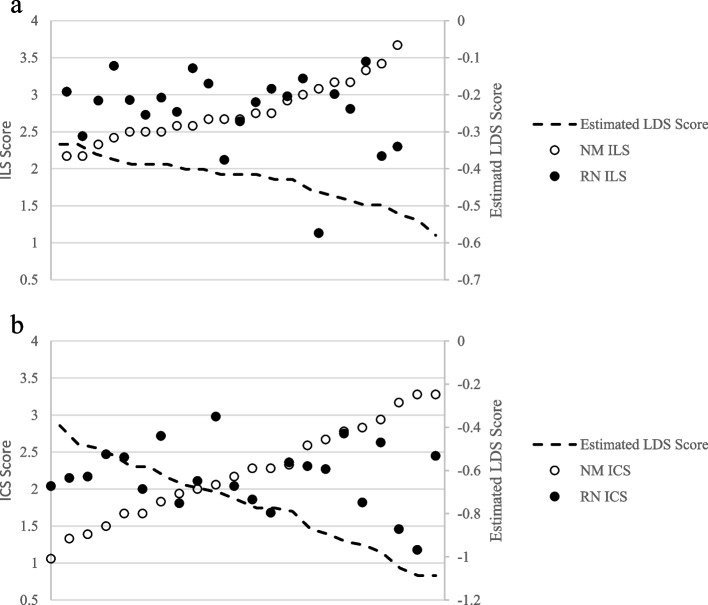
**a** Assessing nurse manager ILS scores on differences in staff-manager ILS scores. **b** Assessing nurse manager ICS scores on differences in staff-manager ICS scores. Note: ILS, Implementation Leadership Scale; ICS, Implementation Climate Scale; RN, registered staff nurse; NM, nurse manager; LDS, latent difference score (predicted)

Tables 2 and 3 show the results from the LDS models. The analyses consistently supported H2a and H2b and found that higher manager ILS and ICS scores were associated with larger differences between staff and managers; as manager scores increased, staff scores increasingly differed with increasingly lower ILS and ICS scores. The variance in the latent difference scores for each ILS subscale and the total score were statistically significant (*p*<.001) and ranged from 0.515 (total score) to .757 (proactive leadership subscale) (see Table 2). In addition, the negative associations between ILS difference scores and manager ILS scores (see Table 2; unstandardized beta weights of manager ILS scores range from −0.164 to −0.385) suggest that as the manager ILS scores increased, the latent difference scores are inverse, and staff scores increasingly differed with increasingly lower ILS scores. The findings are similar for the ICS latent difference scores. The variances in the LDSs for the ICS are statistically significant and range from 0.700 (total ICS score) to 1.31 (educational support for EBP and rewards for EBP subscales) (see Table 3). The relatively large differences (see Table 3 unstandardized beta weights of manager ICS range from −0.313 to −0.788) suggest that as the manager ICS scores increased, the latent difference scores become more negative. Figure 1a and b graphically present this association for both the total ILS and ICS scores. As scores on the manager ILS or ICS total increase, the predicted LDS scores increase from a smaller negative number to a larger negative number, indicating a larger gap between those scores and the lower staff ratings. In conclusion, Hypothesis 2 was supported for all ILS and ICS total and subscale scores.

**Table 2:** Summary of latent difference and crude score models for the ILS (*N* = 22)

	LDS difference^**a**^			Crude difference^**b**^		
	*b*	SE	*p*	b	SE	*p*
**Total ILS score latent difference**						
Nurse manager ILS score	−.164	.053	.002	−1.25	.326	<.001
Years as RN	−.096	.053	.101	−.031	.050	.540
Years as RN on unit	.029	.028	.592	−.009	.055	.870
Education	−1.41	.481	.051	−.396	.702	.580
LDS mean = .022 (SE = .163), p = .890 (baseline model) LDS variance = .515 (SE = .153), p = .011 (baseline model)				Adj. *R*-square=.436		
**Proactive leadership**						
Nurse manager ILS score	−.385	.074	<.001	−1.10	.197	<.001
Years as RN	−.108	.075	.147	−.021	.050	.675
Years as RN on unit	.032	.084	.703	.012	.054	.824
Education	−1.70	.940	.070	−.230	.687	.745
LDS mean = .268 (SE = .206), *p* = .193 (baseline model) LDS variance = .757 (SE = .215), *p* < .001 (baseline model)				Adj. *R*-square=.623		
**Knowledgeable leadership**						
Nurse manager ILS score	−.189	.069	.006	−1.06	.295	.002
Years as RN	−.114	.057	.046	−.037	.054	.501
Years as RN on unit	.112	.049	.023	−.003	.065	.965
Education	−1.29	.676	.055	−.633	.714	.387
LDS mean = .329 (SE = .162), *p* = 042 (baseline model) LDS variance = .576 (SE = .244), *p* = .018 (baseline model)				Adj. *R*-square=.435		
**Supportive leadership**						
Nurse manager ILS score	−.250	.049	<.001	−1.14	.235	<.001
Years as RN	−.092	.059	.118	−.055	.045	.236
Years as RN on unit	−.040	.044	.567	.003	.051	.950
Education	−1.35	.541	.064	−.505	.633	.436
LDS mean = −.300 (SE = .160), *p* = .060 (baseline model) LDS variance = .585 (SE = .176), *p* < .001 (baseline model)				Adj. *R*-square=.567		
**Perseverant leadership**						
Nurse manager ILS score	−.208	.086	.015	−1.22	.310	<.001
Years as RN	−.066	.055	.234	−.041	.052	.445
Years as RN on unit	.013	.056	.820	−.002	.058	.971
Education	−1.17	.737	.110	−.567	.724	.444
LDS mean = −.196 (SE = .177), *p* = .270 (baseline model) LDS variance = .580 (SE = .156), *p* = .002 (baseline model)				Adj. *R*-square=.409		

All models accounted for clustering at the unit and hospital level

*LDS* latent difference score (calculated as staff score minus manager score), *ILS* Implementation Leadership Scale, *RN* registered nurse, *Adj. R-square* adjusted *R*-square

^a^All estimated baseline (not covariates) and fully adjusted models (covariates) are “Just-Identified” (adjusted model fit indices)

^b^Ordinary least squares (OLS) regression (multiple linear regression) was used to model crude difference scores

**Table 3:** Summary of latent difference and crude score models for the ICS (*N* = 22)

	LDS difference^**a**^			Crude difference^**b**^		
	*b*	SE	*p*	*b*	SE	*p*
**Total ICS scale latent difference**						
Nurse manager ICS score	−.313	.170	.003	−1.09	.172	<.001
Years as RN	−.159	.047	<.001	−.056	.041	.186
Years as RN on unit	−.034	.030	.257	.022	.043	.612
Education	−1.82	.729	.013	−.108	.578	.854
LDS mean = −.063 (SE = .195), *p* = .749 (baseline model) LDS variance = .700 (SE = .207), *p* < .001 (baseline model)				Adj. *R*-square=.744		
**Focus on EBP**						
Nurse manager ICS score	−.473	.131	<.001	−1.18	.147	<.001
Years as RN	−.191	.063	.002	.001	.044	.982
Years as RN on unit	−.024	.083	.768	−.009	.042	.828
Education	−2.44	.741	.001	.407	.624	.523
LDS mean = −.077 (SE = .216), *p* = .721 (baseline model) LDS variance = 1.01 (SE = .301), *p* < .001 (baseline model)				Adj. *R*-square=.821		
**Educational support for EBP**						
Nurse manager ICS score	−.488	.143	<.001	−1.18	.145	<.001
Years as RN	−.285	.071	<.001	−.053	.047	.280
Years as RN on unit	−.012	.077	.878	.025	.043	.571
Education	−2.55	.858	.003	−.411	.578	.487
LDS mean = −.085 (SE = .260), *p* = .744 (baseline model) LDS variance = 1.31 (SE = .410), *p* < .001 (baseline model)				Adj. *R*-square=.862		
**Recognition for EBP**						
Nurse manager ICS score	−.619	.190	<.001	−1.07	.123	<.001
Years as RN	−.152	.057	.008	−.043	.040	.289
Years as RN on unit	−.007	.074	.920	.017	.043	.692
Education	−1.63	1.12	.147	−.360	.545	.806
LDS mean = .069 (SE = .221), p = .755 (baseline model) LDS variance = .986 (SE = .234), p < .001) (baseline model)				Adj. *R*-square=.841		
**Rewards for EBP**						
Nurse manager ICS score	−.788	.321	.014	−.937	.121	<.001
Years as RN	−.055	.089	.535	−.084	.045	.078
Years as RN on unit	−.037	.064	.557	.048	.052	.368
Education	−.765	.857	.372	−.133	.618	.832
LDS mean = .230 (SE = .225), *p* = .307 (baseline model) LDS variance = .988 (SE = .343), *p* = .012 (baseline model)				Adj. *R*-square=.740		
**Selection for EBP**						
Nurse manager ICS score	−.646	.170	<.001	−1.13	.133	<.001
Years as RN	−.179	.074	.015	−.069	.043	.126
Years as RN on unit	.036	.052	.493	.029	.046	.533
Education	−2.38	1.03	.020	.030	.627	.963
LDS mean = .149 (SE = .254), *p* = .558 (baseline model) LDS variance = 1.14 (SE = .299), *p* < .001 (baseline model)				Adj. *R*-square=.812		
**Selection for openness**						
Nurse manager ICS score	−.414	.104	<.001	−1.08	.166	<.001
Years as RN	−.057	.058	.332	−.050	.041	.240
Years as RN on unit	.086	.056	.124	−.008	.049	.871
Education	−.903	.950	.342	−.073	.580	.902
LDS mean = −.297 (SE = .180), *p* = .099 (baseline model) LDS variance = .683 (SE = .174), *p* < .001 (baseline model)				Adj. *R*-square=.678		

All models accounted for clustering at the unit and hospital level

*LDS* latent difference score (calculated as staff score minus manager score), *ICS* Implementation Climate Scale, *RN* registered nurse, *Adj. R-square* adjusted *R*-square

^a^All estimated baseline (not covariates) and fully adjusted models (covariates) are “Just-Identified” (adjusted model fit indices)

^b^Ordinary least squares (OLS) regression (multiple linear regression) was used to model crude difference scores

Although Hypothesis 3 stated increased staff RN experience will be associated with less perceptual difference, the opposite was found. The average number of years as an RN among staff on study units was found to be associated with larger differences in only ILS knowledgeable leadership score, the ICS total score, and most ICS subscale scores (except for rewards for EBP and selection for openness) (see Tables 2 and 3). As the average number of years as an RN on a unit increased among staff, the difference scores increased, with staff ILS and ICS scores becoming increasing lower than their manager scores as RN years increased (see Supplemental file 2 for graphical depiction).

Hypothesis 4 stated that greater years of staff tenure on the unit as an RN would be associated with less perceptual distance. The analysis found that average tenure on the unit was associated with less perceptual distance for only knowledgeable leadership. Finally, it should be noted that although not hypothesized, the confounder of average education level of staff on the study unit was found to be negatively associated the ICS total score and half of ICS subscale scores (focus on EBP, educational support for EBP, selection for EBP). In other words, high levels of education on the unit were associated with greater perceptual differences. Hypothesis 4 was partially supported.

**Fig. 2:**
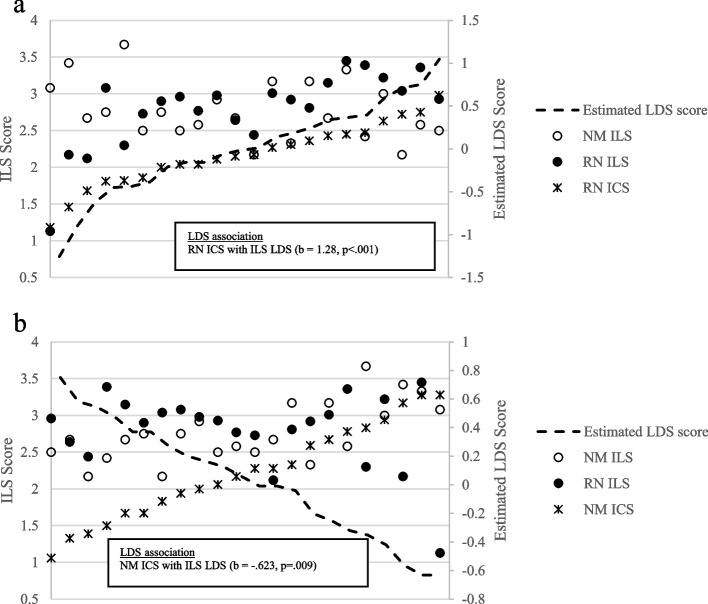
**a** Assessing the association with differences in staff-manager ILS scores with staff ICS scores. **b** Assessing the association with differences in staff-manager ILS scores with manager ICS scores. Note: ILS, Implementation Leadership Scale; ICS, Implementation Climate Scale; NM, nurse manager; RN, registered staff nurse; LDS, latent difference score (predicted)

Figure 2 shows that the association between the ILS LDS (perceptual difference between staff and managers) and staff ICS scores was statistically significant and indicated a strong positive association (*b* = 1.28, *p* < .001). Figure 2a illustrates this association; as the latent difference ranged from manager ILS scores being higher than staff scores (i.e., negative LDS values) to staff scores being higher than manager scores (i.e., positive values), staff ICS perceptions increased accordingly. Thus, Hypothesis 5 was partially supported because we did not anticipate staff scores being higher than manager scores. Although not hypothesized, we also examined this relationship for nurse manager ICS scores and found the opposite. A negative association was found between ILS LDS scores and manager ICS scores (*b* = −.623, *p* = .009); see Fig. 2b. As the latent difference ranged from manager ILS scores being higher than staff scores (i.e., negative LDS values) to staff scores being higher than nurse manager scores (i.e., positive values), nurse manager ICS perceptions decreased accordingly, such that when staff rated the managers higher than the managers rated themselves, the ICS scores were lowest.

## Discussion

This study is the first to describe perceptual differences between nurse managers and staff nurses regarding leadership and climate for implementation. Differences in leadership and climate for implementation aggregated across units by role (H1)—manager versus staff—were few but staff rated managers higher on knowledgeable leadership. Managers are expected to maintain competency in EBP and support staff efforts to implement EBP [18, 46]; however, studies have found that nurse managers lack sufficient knowledge and competency related to EBP implementation [42, 47]. Their self-perceived lack of knowledge may explain why they scored themselves lower than staff. In contrast, managers’ ratings of supportive leadership were higher than staff. One possible explanation for lower ratings by staff is that nurse managers often delegate the leadership for activities to implement EBPs and education of staff to advanced practice nurses (e.g., clinical nurse specialist; nurse educator) [48]. Although hiring and maximizing use of the knowledge and skills of advanced practice nurses regarding EBP are a supportive behavior of managers, staff may not consider the role and efforts of these advanced practice nurses as an extension of managerial support. More research is needed to understand how nurse manager factors (e.g., experience, tenure, education) and unit factors (e.g., clinical nurse specialist on staff) affect their ratings of leadership and climate and the difference of these ratings by staff.

Despite the general lack of difference between manager and staff ratings aggregated across units, analyses at the unit level provided a more nuanced understanding of the differences between managers and staff. For all measures, as managers’ scores increased, the latent difference scores (LDS) also increased; that is the difference between the manager and staff ratings widened. Findings from Hypothesis 2 provide some support for the Dunning-Kruger effect [31] and/or the argument that access to information may lead to higher manager ratings [33]. However, this study could not differentiate which theory may explain the observed differences. Further, it is unclear which group, managers or staff, had the most accurate rating of leadership and climate, which can vary by unit. Some managers, in line with the Dunning-Kruger effect, may have the tendency to rate themselves higher than their actual performance and rate the climates of the units they create more positively than they are in reality, which results in an inaccurate rating. Others may rate higher due to access to information and a better understanding of their own behaviors and work environments they create, lending to a potentially more accurate rating. And still others may be rating higher for both reasons. Regardless, staff perceptions, particularly of climate, are critical to consider as previous research has found perceptions of climate to be related to unit performance and strategic success [22].

A key component for creating a strategic climate for implementation is managers effectively communicating what is prioritized and valued to the staff. Thus, one potential mechanism to decrease perceived difference between managers and staff regarding unit climate for EBP implementation is to increase the amount and type of communication around implementation efforts. If perceptual differences are being driven by a self-serving bias of nurse managers, then increased feedback from staff will help them have a more realistic view of their own leadership and the climate of their unit. Alternatively, if perceptual differences are being driven by accessibility to information, then increased communication will promote awareness by staff of efforts by their managers to lead and support implementation such as access to knowledge and skills of experts, hiring staff and advanced practice nurses with expertise in EBP, and including monetary support in unit budgets for staff education thereby fostering positive implementation contexts. Failure to effectively communicate these efforts may not only result in greater perceptual discrepancy, but also low utilization of EBP resources that could improve implementation. Further, effective communication has been linked to creating healthy nursing work environments and retaining and engaging staff [49–51] and has been described as a core competency for leading staff during times of crisis and rapid implementation [43]. The results for Hypothesis 5 were mixed on the implications for perceptual differences on leadership for climate depending on whether the outcome was staff or manager perceptions of climate; thus, more aligned perceptions may split the difference and result in the optimal climate.

Total number of years of experience as a registered nurse for staff was associated with perceptual differences on many of the climate domains, including the total climate score, and one of the leadership domains (knowledgeable leadership). Atwater and Yammarino [40] proposed that individuals with more tenure or job experience rate others higher than raters with less experience. Although this was not supported for total years of nursing experience, which includes experience in different units and organizations, it was supported for staff’s average tenure on the unit for the leadership domain of knowledge. Staff with greater exposure to their managers may be better positioned to accurately rate their manager’s knowledge about EBP and implementation. The associations among total years of RN experience and climate and leadership measures were inverse and opposite of what we hypothesized. It is possible that our sample included staff with many years of overall experience but few years of experience on the study unit. Further, this finding may be explained by relatively high staff attrition rates on medical-surgical nursing units [52].

### Limitations

This study has limitations. First, a relatively small convenience sample of hospitals, units, and participants was used. This limitation was minimized by recruiting hospitals of various size, ownership, and location. For participants, 30 eligible staff on each unit were randomly selected to receive invitations to participate to limit response bias. Our study only included a staff perspective of leadership and climate and compared this perspective to the nurse managers’ perspective. Including and comparing other perspectives (e.g., the nurse managers’ supervisors, physicians, clinical nurse leaders) may be a direction for future research. There is the possibility of common method bias in this study as the ILS and ICS were completed at the same time by the same sample; however, such issues were not expected to substantially affect the findings due to the focus on perceptual differences. The current study did not include manager tenure, which may be a potential factor explaining differences in perceptions and thus should be addressed in future research.

## Conclusions

Implementation leadership and climate are important social dynamic factors affecting the implementation of EBPs into clinical practice. Perceptual differences between nurse managers and staff may signal relationship and communication issues within the unit which could ultimately affect implementation success. Prior to implementation of EBP in nursing settings, we encourage exploring manager and staff perceptions of implementation leadership and climate and identify areas of difference. These areas, along with domains in which both managers and staff agree are low, provide potential targets for implementation strategies and interventions.

## Supplementary Information

**Additional file 1.** Correlation Matrix (*N* = 22).

**Additional file 2.** Differences in ICS Scores and Average Years as an RN.

## Data Availability

Data and materials are available from the corresponding author on reasonable request.

## Abbreviations

ILS: Implementation Leadership Scale
ICS: Implementation Climate Scale
LDS: Latent difference score
EBP: Evidence-based practice
RN: Registered nurse
CFI: Comparative Fit Index
TLI: Tucker Lewis Index
RMSEA: Root Mean Square Error of Approximation
