# Supplementary material for: Perceptual differences in nursing implementation leadership and climate: a cross-sectional study
Source: Implement Sci Commun. 2023 Jan 20;4:9. doi: 10.1186/s43058-023-00392-9 (PMC9854059; doi:10.1186/s43058-023-00392-9)
Supplement: Supplementary file 1 — Additional file 1. Correlation Matrix (N = 22). [file 43058_2023_392_MOESM1_ESM.docx]

| **Supplementary File 1: Correlation Matrix (N=22)** | | | | | | |
| --- | --- | --- | --- | --- | --- | --- |
|  | **NM-ILS** | **NM-ICS** | **RN-ILS** | **RN-ICS** | **Years as RN** | **Years as RN on Unit** |
| **NM-ILS** |  |  |  |  |  |  |
| **NM-ICS** | .664** |  |  |  |  |  |
| **RN-ILS** | -0.251 | -0.268 |  |  |  |  |
| **RN-ICS** | -0.372 | -0.208 | .785** |  |  |  |
| **Years as RN** | 0.071 | 0.201 | -0.124 | -0.404 |  |  |
| **Years as RN on Unit** | -0.086 | 0.298 | -0.078 | -0.072 | 0.354 |  |
| **Education** | 0.221 | 0.232 | -0.085 | 0.189 | -.646** | -0.124 |
| ** Correlation is significant at the 0.01 level (2-tailed). NM= Nurse manager; ILS= Implementation Leadership Scale; ICS= Implementation Climate Scale; RN= Registered nurse | | | | |  |  |

Shuman, Ehrhart, Veliz, & Titler: Perceptual differences in nursing implementation leadership and climate: a cross-sectional study
