# Supplementary material for: Perceptual differences in nursing implementation leadership and climate: a cross-sectional study
Source: Implement Sci Commun. 2023 Jan 20;4:9. doi: 10.1186/s43058-023-00392-9 (PMC9854059; doi:10.1186/s43058-023-00392-9)
Supplement: Supplementary file 2 — Additional file 2. Differences in ICS Scores and Average Years as an RN. [file 43058_2023_392_MOESM2_ESM.docx]

Note: ICS= Implementation Climate Scale; NM= Nurse Manager; RN= Registered Staff Nurse; LDS= Latent Difference Score (Predicted)
